# Cryptosporidiosis — A Plausible Cause for Relapse of Guillain-Barré Syndrome

**DOI:** 10.7759/cureus.14652

**Published:** 2021-04-23

**Authors:** Asadullah Anees Khan, Karthik Somasundaram

**Affiliations:** 1 Intensive Care Unit, St. Peter's Hospital, Ashford and St. Peter's Hospitals NHS Trust, Chertsey, GBR

**Keywords:** guillain barre syndrome (gbs), guillain-barre syndrome (gbs), acute flaccid paralysis, bilateral limb weakness, cryptosporidiosis, gastroenteritis, id critical care, traveller medicine, relapse, neurology and critical care

## Abstract

A 25-year-old female presented on the acute medical take with rapidly evolving ascending weakness, sensory loss, and areflexia after a prodromal diarrhoeal illness, ultimately critical care admission, tracheostomy, and intravenous immunoglobulin (IVIG) therapy. The patient had been diagnosed with Guillain-Barré Syndrome (GBS) six years previously, treated with intravenous Immunoglobulin, and discharged after a five-day in-patient stay without mechanical ventilation. On this occasion, a diagnosis of recurrent GBS was made, supported by cytoalbuminological dissociation in the cerebrospinal fluid (CSF). Investigations for infective precipitants were negative aside from a stool culture, positive for *Cryptosporidium spp*. DNA (deoxyribonucleic acid) two weeks earlier. There are no previously reported cases of GBS due to cryptosporidiosis on PubMed.

The patient was treated with a course of IVIG and discharged from critical care after 66 days, requiring ongoing neurorehabilitation, which is likely to be prolonged.

## Introduction

Guillain-Barré Syndrome (GBS) is a well-recognised demyelinating polyneuropathy that is preceded by a prodromal illness in two-thirds of cases. Recurrent episodes are uncommon but occur in a subset of patients, being commoner in patients below 30 years of age [1]. Causative organisms include *Campylobacter jejuni*, Hepatitis viruses, *Cytomegalovirus *(CMV), *Mycoplasma*, Epstein-Barr virus (EBV), *Haemophilus influenzae,* and others [2,3]. Our literature review identified that cryptosporidiosis has not previously been identified as a precipitant of GBS.

## Case presentation

A 25-year-old female presented with distal sensory loss in the upper limbs and gradual onset progressive ascending weakness over 3-4 days.

She suffered from a diarrhoeal illness recently, having returned from Pakistan four weeks before presentation to hospital. Stool cultures sent from primary care two weeks before admission returned positive for *Cryptosporidium spp.* DNA (deoxyribonucleic acid) polymerase chain reaction (PCR) and she was treated with ciprofloxacin for this. Testing for common infective precipitants including *Campylobacter *was carried out on the same sample however returned a negative result. Her presenting symptoms followed a paediatric admission for her 13-month-old daughter’s following a similar bout of gastroenteritis. The daughter did not undergo stool culture testing and no precipitating organism was identified in her case.

The patient had experienced an episode of acute Guillain-Barré Syndrome six years previously with rapid recovery at another institution. On that occasion, she required one course of intravenous immunoglobulin (IVIG) leading to improvement and never experienced respiratory failure. The only other past medical history of note was gestational diabetes mellitus.

On examination, she had symmetric ascending flaccid paresis with weakness and areflexia. Flexion and extension movements at the hip, knee, and ankle were MRC (Medical Research Council) Grade 2/5 bilaterally with MRC Grade 3/5 at the wrist and 4/5 at the elbow as well as the shoulder. The total MRC-sum score on admission was 36/60. On presentation, due to a lack of facial weakness, she had a total EGRIS (Erasmus GBS Respiratory Insufficiency Score) score of 4 which predicted a 32% chance of respiratory insufficiency during the first week of admission. She was physiologically stable when admitted, however, was moved to critical care for monitoring of FVC (forced vital capacity). Twelve hours into her admission, she developed a weak voice and with a rising oxygen requirement and was subsequently intubated and mechanically ventilated for progressive respiratory failure.

Admission blood tests were within normal limits, however, the inflammatory markers (white cell count and C-reactive protein) proceeded to rise in the first two days of admission. Treatment was instituted for aspiration pneumonia due to the visualisation of pooled saliva in the airway pre-intubation. Nasogastric feeding (NG) was commenced as a prolonged period of ventilation was anticipated and the patient had already demonstrated cranio-bulbar signs.

A CT Brain on day 1 of her admission did not reveal any abnormalities and she underwent full neuraxial imaging on day 3 of admission including magnetic resonance scanning of the brain and spinal cord with contrast - these did not reveal an abnormal pathology either and were within normal limits. She underwent a lumbar puncture on day 1 of admission which revealed evidence of cyto-albuminological dissociation (Table 1).

**Table 1:** Lumbar Puncture Results CSF - cerebrospinal fluid

Test	Result	Range
Appearance	Clear, colourless	
Red blood cells (last sample)	<1 x 10^6^/L	
White blood cells	<1 x 10^6^/L	
Organisms	No organisms seen	
Culture	No growth after 48 hours	
Protein	0.53 g/L	0.15 – 0.4
Oligoclonal bands Albumin (CSF)	0.37 g/L	0.00 – 0.17
Oligoclonal bands IgG (CSF)	0.04 g/L	0.000 – 0.017

She was reviewed by the neurologist and investigations for HIV, hepatitis viruses, Epstein-Barr virus (EBV), *Cytomegalovirus *(CMV), ANA (Hep-2), GM1 Abs, GQ1B Abs, Anti-MPO, and Anti-PR as well as Lyme disease and syphilis were negative. Nerve conduction studies were performed and demonstrated in-excitability (described as absent distal motor responses or only present in one nerve with very low amplitude) of peripheral motor nerves and normal sensory nerves, in keeping with the Guillain-Barré Syndrome spectrum.

Treatment was commenced with IVIG at a dose of 0.4 kg/bodyweight for five days. This was followed by an IVIG course two weeks after the first course. A tracheostomy was sited on day 4 of admission. She remained in critical care for 66 days until she had demonstrated sufficient improvement in peripheral muscular function and recovery of strength in her pharyngeal muscles. Her admission was complicated by low mood and neuropathic pain which were treated with citalopram and pregabalin respectively. She was discharged to the ward after decannulation, and the NG tube was removed after being cleared by the SALT (Speech and Language Team) with ongoing physiotherapy support. Her recovery is expected to be prolonged.

Three weeks after moving to the respiratory ward, she was discharged to a neuro-rehabilitation unit where she stayed for two months. She has now been discharged to her own home, where she is still improving and has regained use of her upper limbs, walking with crutches. She still requires regular physiotherapy.

## Discussion

This is the first reported trigger of GBS secondary to cryptosporidiosis. Relapse of GBS is not common - reportedly only 1 - 3% of cases [4]. Relapses are also milder than the original illness, common in patients below 30 years of age, and usually Miller-Fisher variants. [5] Recurrent GBS is also not necessarily associated with a prodrome and can be resistant to immunomodulatory treatment as in this case where the patient required two courses of IVIG and without rapid or complete resolution of symptoms to date.

There are four main mechanisms thought to lead to an immune response responsible for the signs and symptoms of Guillain-Barré Syndrome [6]: 1. *Antibody mediated *- Serum antibodies to peripheral nerves (Anti-GM1, Anti-GD1a, Anti-G1b, etc.) are demonstrable in 50% of patients [6]; 2. *Molecular mimicry *- The most likely mechanism due to evidence of *Campylobacter *infection leading to GBS, certain lipo-oligosaccharides react with gangliosides in nervous tissue, these anti-bodies are also cross-reactive [6]; 3. *Complement mediated* - Due to complement activation at the nerve site. There is some evidence for the complement inhibitor eculizumab inhibited neurotoxicity [6]; 4. *Patient susceptibility* - Certain factors in individuals might make them more susceptible to infection, therefore increasing the risk of developing GBS.

*Cryptosporidium *is a parasitic alveolate that is known to cause mostly gastrointestinal, biliary, and respiratory infections in humans [7]. This parasite has a peculiar ability to colonise many epithelial surfaces and has been identified in the pancreas as well as the urinary bladder as mentioned by Sponseller et al. [7]. This is more common in immunodeficient hosts (e.g., with HIV) [8], but it can also cause a self-limiting illness in the immunocompetent [8]. There are still 4000 cases annually in England and Wales despite a reduction in reported cases [9]. The illness can spread by contacting infected animals or drinking contaminated water. In developing countries, 8-19% of diarrhoeal illnesses can be attributed to cryptosporidiosis [10].

There is a plausible case for *Cryptosporidium parvum *to be responsible for immunologic phenomena in humans. As we know, *Campylobacter jejuni *exhibits molecular mimicry [11] - the lipopolysaccharides on *C. jejuni *react with gangliosides in nerve cells causing the formation of anti-ganglioside antibodies (which are detectable in sera of patients). This molecular mimicry leads to neuronal destruction and subsequent neurological weakness. Some reports have suggested cases of GBS associated with *Campylobacter *have a worse prognosis due to the severity of the autoimmune response causing axonal injury [11,12]. In a similar vein, antibodies in humans and animals with cryptosporidiosis react with antigenic proteins (CApy and CP15) on the organism’s surface, making an immunological response likely. Currently, this has only been studied experimentally in mice models [13]; further research and awareness are needed in human hosts. These same antigenic targets are being investigated in order to develop a vaccine. 

*This episode of neurological weakness is GBS and not CIDP (chronic inflammatory demyelinating polyneuropathy) due to the following reasons:* There was an acute course preceded by prodromal illness (progression over days as opposed to weeks and months which is common in CIDP); the presence of respiratory failure - this is uncommon in CIDP as opposed to classic GBS (AIDP, etc.); improvement with IVIG and no requirement for steroid therapy; as well as no previous relapses since the initial episode five years ago.

Extra-intestinal manifestation of *Cryptosporidial *infection has been previously described. Several studies have shown that infection with *Cryptosporidium hominis *is linked to extra-intestinal sequelae like arthralgias, ocular symptoms, dizziness, headache, and malaise [14].

## Conclusions

To conclude we would like to highlight the following points: *Cryptosporidium *has not been known to cause Guillain-Barré Syndrome but should be recognized as a potential trigger. Patients presenting with weakness and reduced/absent tendon reflexes with a prodromal infective aetiology should be tested for cryptosporidiosis if usual precipitants are not identified - diarrhoeal illnesses that cause GBS may be undiagnosed cryptosporidiosis. A history of previous GBS does not rule out further relapses of GBS. Although uncommon, GBS can recur at any point with variable sequalae.
